# Persistent muscle hyperalgesia after adolescent stress is exacerbated by a mild-nociceptive input in adulthood and is associated with microglia activation

**DOI:** 10.1038/s41598-022-21808-x

**Published:** 2022-10-31

**Authors:** Sathish Kumar Singaravelu, Alexander Dawit Goitom, Akseli Petteri Graf, Handan Moerz, Andreas Schilder, Ulrich Hoheisel, Rainer Spanagel, Rolf-Detlef Treede

**Affiliations:** 1 Department of Neurophysiology, Mannheim Center for Translational Neuroscience, Medical Faculty Mannheim, Heidelberg University, Ruprecht-Karls-University Heidelberg, Ludolf-Krehl-Str. 13-17, 68167 Mannheim, Germany; 2 Medical Faculty Mannheim, Institute of Psychopharmacology, Central Institute of Mental Health, Heidelberg University, Mannheim, Germany; 3 Department of Experimental Orthopedics and Trauma Surgery, Medical Faculty Mannheim, Heidelberg University, Mannheim, Germany

**Keywords:** Molecular biology, Neuroscience

## Abstract

Non-specific low back pain (LBP) is a major global disease burden and childhood adversity predisposes to its development. The mechanisms are largely unknown. Here, we investigated if adversity in young rats augments mechanical hyperalgesia and how spinal cord microglia contribute to this. Adolescent rats underwent restraint stress, control animals were handled. In adulthood, all rats received two intramuscular injections of NGF/saline or both into the lumbar multifidus muscle. Stress induced in rats at adolescence lowered low back pressure pain threshold (PPT; *p* = 0.0001) and paw withdrawal threshold (PWT; *p* = 0.0007). The lowered muscle PPT persisted throughout adulthood (*p* = 0.012). A subsequent NGF in adulthood lowered only PPT (*d* = 0.87). Immunohistochemistry revealed changes in microglia morphology: stress followed by NGF induced a significant increase in ameboid state (*p* < 0.05). Repeated NGF injections without stress showed significantly increased cell size in surveilling and bushy states (*p* < 0.05). Thus, stress in adolescence induced persistent muscle hyperalgesia that can be enhanced by a mild-nociceptive input. The accompanying morphological changes in microglia differ between priming by adolescent stress and by nociceptive inputs. This novel rodent model shows that adolescent stress is a risk factor for the development of LBP in adulthood and that morphological changes in microglia are signs of spinal mechanisms involved.

## Introduction

Low back pain (LBP) is a major cause of suffering and disability^1,2^. Especially non-specific LBP is a global burden due to its high prevalence and persistent interference with daily life^3,4^. In ICD-11 (International Classification of Diseases), it is considered as an important problem in medical care in its own right^5^. Adolescence is a critical period in the human development and adverse childhood experiences (ACEs) have long-term adverse effects on both mental and physical health^6^. Specifically, adversities in earlier life such as emotional abuse are associated with enhanced temporal summation of pain and sexual abuse with enhanced touch sensitivity^7^. In humans, stress and ACEs are risk factors for the chronicity of subacute LBP^8^ and the development of chronic widespread pain^9^ yet their underlying mechanisms remain elusive.

Experimental animal models mimic several human pain conditions including neuropathic and musculoskeletal pain^10^. In adult rats, mild-nociceptive input induced by a single nerve growth factor (NGF) injection into the low back muscle can induce a state of latent sensitization in dorsal horn neurons (DHNs) that primes the neurons to be more easily sensitized by a subsequent NGF injection. The resulting manifest sensitization is characterized by mechanical hyperalgesia in the low back, a significant increase in resting activity, and the appearance of new receptive fields in deep tissues extending into the hind limb^11^. In this model, the two NGF injections mimic repeatedly injured or overloaded muscles^12–14^. NGF is released by normal muscle cells and this release is potentiated by muscle injury^13,15^. In humans, intramuscular NGF injections induce a long-lasting mechanical hyperalgesia^16,17^. Furthermore, inhibiting NGF signaling may alleviate pain in chronic LBP patients^18^.

We previously showed that stress in adulthood can prime DHNs^19^ and a subsequent NGF injection leads to a manifest sensitization^20^, suggesting that stress by itself may induce latent sensitization to a subsequent mild nociceptive input. Because adolescence is a more stress sensitive period where drastic changes of neuronal architecture and function occur that lead to distinct behavioral alterations^21^, we expect stress during this period to have a larger effect on DHN priming. Insults across adolescence in rodents induce robust and long-lasting effects on pain related behavior, fear behavior and expression of neuroinflammatory mediators^22–25^.

Latent sensitization induced by NGF injection is mediated by spinal cord microglia activation^26^ via the fractalkine pathway^27^. Activated microglia express ionized calcium-binding adapter molecule 1 (Iba-1); as the Iba-1 marker increases, the morphology of microglial cells changes from ramified to ameboid shapes with enlarged cell bodies and shortened processes. Activated microglia produce various pro-inflammatory cytokines that facilitate the excitatory synaptic transmission in the spinal cord^28^, leading to pain hypersensitivity^29–31^. Several studies have also shown morphological changes of microglia in chronic stress^32,33^.

Here, we aimed to study the long-term effect of stress during adolescence on microglia states and the propensity for DHN sensitization and their potential neurobiological mechanisms. For this purpose, we compared latent vs. manifest sensitization induced by injections of NGF (as a model of mild nociceptive input) or adolescent restraint (as a model of childhood adversities) or both. We applied combinations of three interventions: restraint stress or control handling (R or C) followed by two injections of NGF or saline (N or S) or both. We investigated four groups: RSS and CSN were predicted to induce latent sensitization (microglia activation but no behavioral signs), while RSN and CNN were predicted to induce manifest sensitization (microglia activation plus signs of hyperalgesia). We evaluated stimulus-evoked pain-related behaviors after each intervention followed by immunohistological analysis of Iba-1 staining intensity and morphological changes in individual microglia cells in lumbar (L2) spinal cord sections.

## Results

### Pressure pain threshold after stress

**Figure 1:**
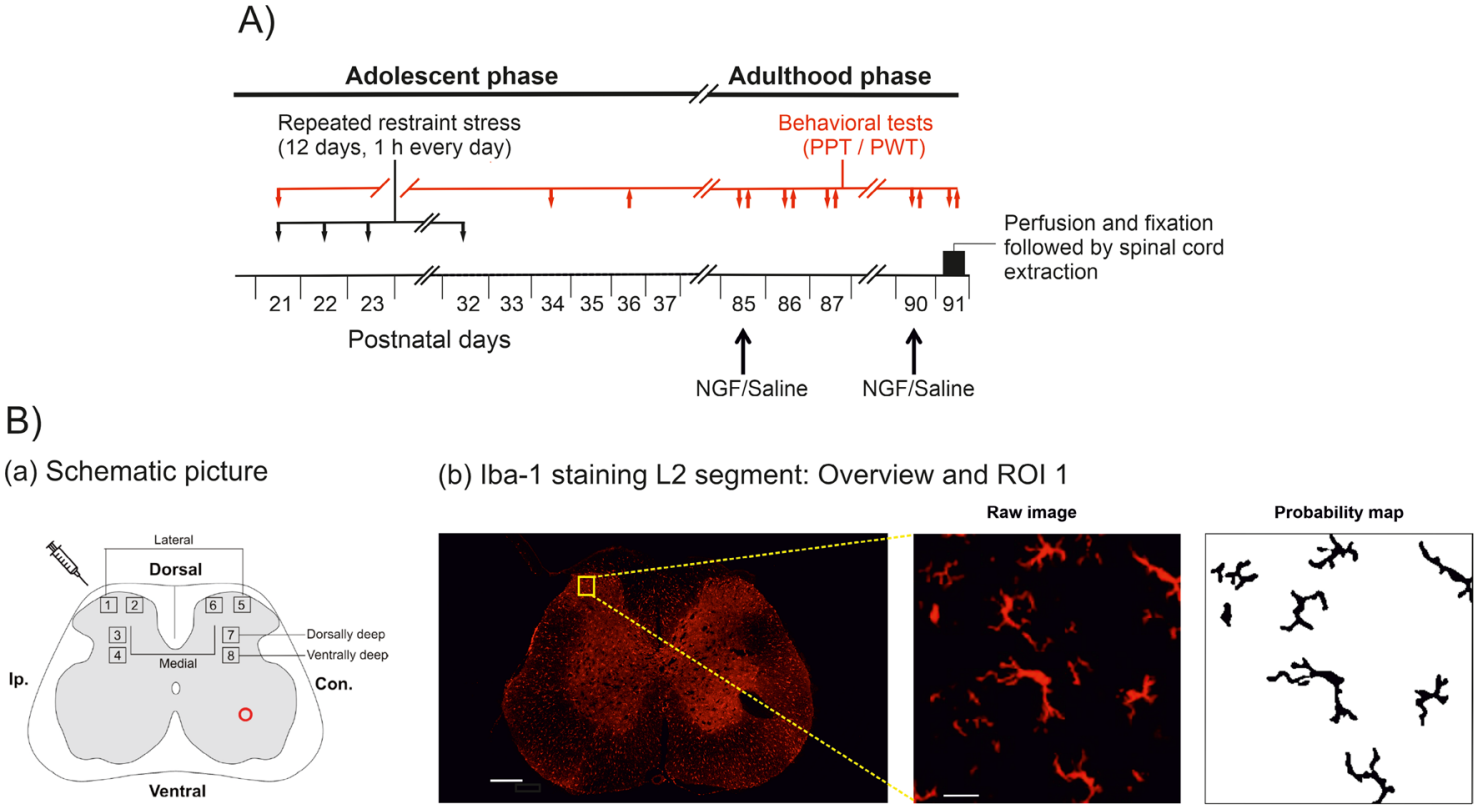
Experimental procedure. (**A**) Animals of the stress group were repeatedly stressed (black line) in a narrow plastic restrainer for 12 consecutive days for 1 h every day on postnatal days (PD21-32; early adolescent phase; black line). The pressure pain threshold (PPT) of the left multifidus muscle at vertebral level L5 and paw withdrawal threshold (PWT) of the left hind paw was measured at different time points (red line) (PPT—downward arrow: PD21, PD34, PD85, PD86, PD87, PD90, PD91; PWT—upward arrow: PD36, PD85, PD86, PD87, PD90, PD91). All animals received injections of NGF/saline on PD85 and PD90 (adulthood phase) according to the group they belonged to (refer to section Treatment groups). On PD91 (black bar) all animals were perfused and fixed. The spinal cord tissues were extracted and stored. (**Ba**) A schematic illustration of the regions of interest on a spinal lumbar 2 (L2) section (refer to section Image processing). The red circle on the ventral horn of the contralateral side denotes the manually made pinhole to identify the contralateral side. (**B.b**) An example overview image (10x) of an Iba-1 stained L2 section (scale bar: 200 µm, background subtracted) and magnified (40x) of ROI 1 (scale bar: 10 µm, Raw image and Probability map). The hole on the ventral horn of the contralateral side denotes the manually made hole with a pin for the identification of contralateral side. PD, postnatal day; Ip., ipsilateral; Con., contralateral; Iba-1, ionized calcium binding adapter molecule 1; ROI, region of interest.

**Figure 2:**
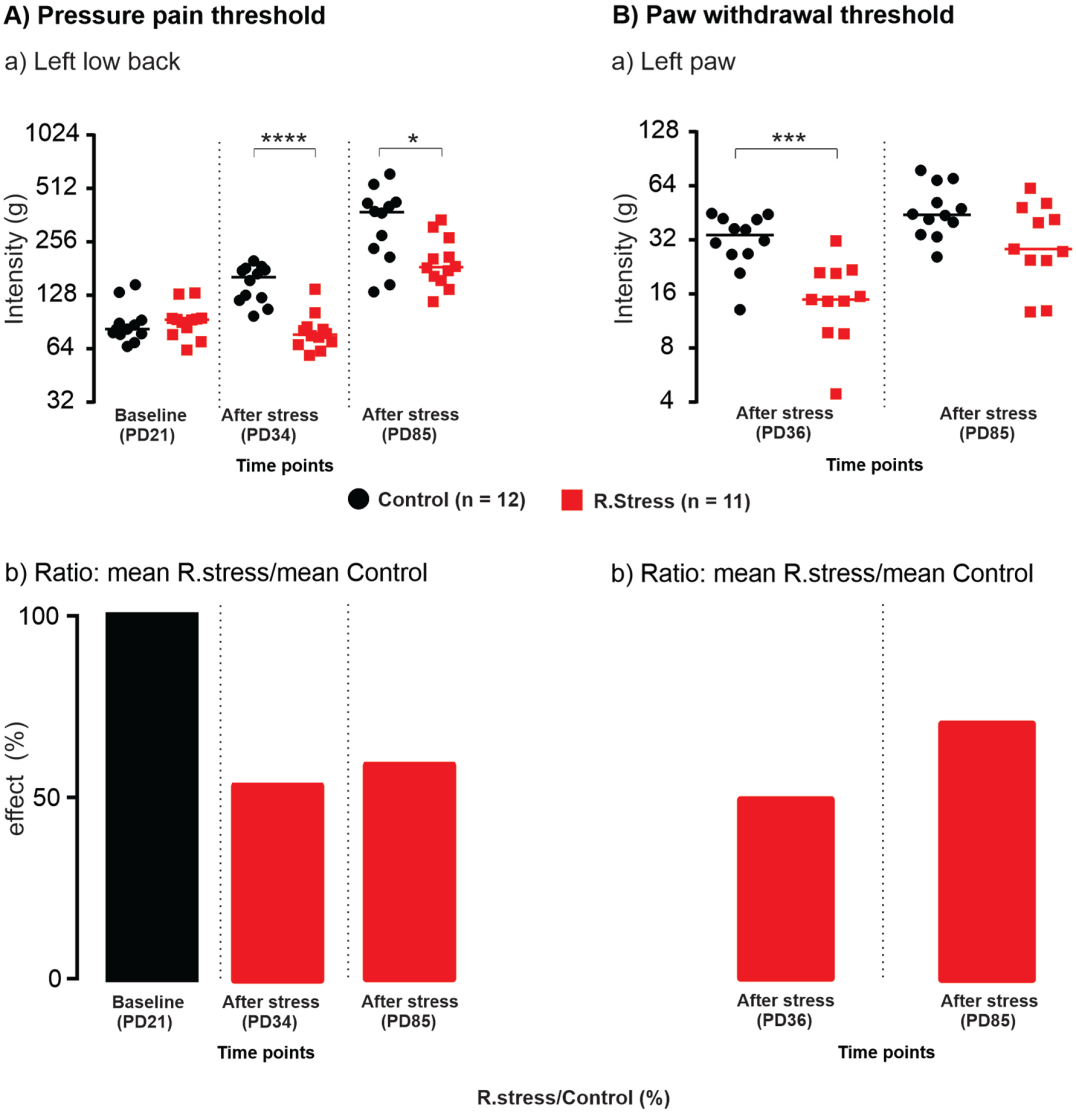
Pressure pain threshold and paw withdrawal threshold before and after stress. Repeated restraint stress in early adolescence induces long-lasting local muscle hyperalgesia and remote cutaneous hyperalgesia compared with non-stressed controls. (**A.a**) Individual data points expressed in log scale, force (in ‘g’ on the left y-axis) required to elicit a pain-related reaction (withdrawal behavior, escape movements, vocalization) using a blunt probe with an area of 3.46 mm^2^ when applied to the left multifidus muscle of the low back. Horizontal lines indicate the median for each group. (**A.b**) Ratio (in ‘%’) of mean pressure pain threshold R.stress/control. (**B.a**) Individual data points expressed in log scale, force (in ‘g’ on the left y-axis) required to elicit a pain-related reaction (paw licking, paw withdrawal) using a rigid cylindrical tip with an area of 0.8 mm^2^ when applied on the plantar surface of the left hind paw. Horizontal lines indicate the median for each group. (**B.b**) Ratio (in ‘%’) of mean paw withdrawal threshold R.stress/control. PD, postnatal days (see Fig. 1A). *P*-values: *U*-test of Mann and Whitney; *P* < 0.05 is represented with (*), *P* < 0.006 (**), *P* < 0.0001 (***), and *P* < 0.0001 (****), asterisks (*) represent the difference between the groups (stress vs. control).

The baseline measurement for the PPT of the left low back multifidus muscle at postnatal day 21 (PD21, Fig. 1A) revealed no significant difference between the control and stress groups (Fig. 2.A.a, *U* = 54, n_1_ = 12, n_2_ = 11, *P* = 0.3186). Two days after the stress paradigm (PD34), the PPT was significantly lower than in the control group (Fig. 2Aa; *U* = 6, n_1_ = 12, n_2_ = 11, *P* < 0.0001), suggesting that the stress paradigm induced axial muscle hyperalgesia. This sensitization was still significant (*U* = 27.50, n_1_ = 12, n_2_ = 11, *P* = 0.0164) in adulthood (PD85). Stress-induced changes were superimposed over a general maturational trend towards higher thresholds over time^34^, hence stress-induced changes are illustrated best by the ratios between stressed and control groups (Fig. 2Ab), which were 100% at baseline, 50% on PD 34 and 60% two months later. These data suggest that the stress paradigm induced a long-term sensitization to the axial muscle input.

### Paw withdrawal threshold after stress

Baseline measurement could not be performed for the PWT. Four days after the stress paradigm (PD36), the PWT of the left hind paw was significantly lower (*U* = 14, n_1_ = 12, n_2_ = 11, *P* = 0.0007) in the repeated restraint stress group than in the control group (Fig. 2Ba) and the ratio was 50% (Fig. 2Bb), suggesting that the stress paradigm induced also distal cutaneous mechanical hyperalgesia. This sensitization was attenuated in the adulthood phase (PD85) (*U* = 34, n_1_ = 12, n_2_ = 11, *P* = 0.0512; Fig. 2Ba) and the ratio between stressed and control groups increased from 50% on PD36 to 70% on PD85 (Fig. 2Bb). These data suggest that the stress paradigm induced a short-term sensitization to the distal cutaneous input.

### Pressure pain threshold of the left low back after NGF/saline injections

In each animal intramuscular injections of NGF or saline were made as a second and third intervention to induce spinal sensitization.

**Figure 3:**
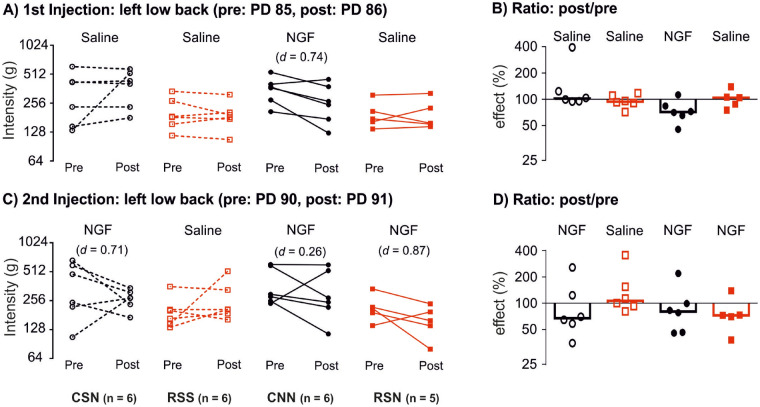
Pressure pain threshold before and after saline/NGF injections. Intramuscular NGF injection leads to an increased pain-related behavior when preceded by repeated restraint stress. (**A**) First injection, pair-wise comparison of individual data points pre and post injection expressed in log scale, force (in ‘g’ on the left y-axis) required to elicit a pain-related reaction (withdrawal behavior, escape movements, vocalization) using a blunt probe with an area of 3.46 mm^2^ when applied to the left multifidus muscle of the low back. (**B**) Individual data points in ratio (in ‘%’), change of pressure of pain threshold shown as effect post/pre. Bars indicate the median for each group. (**C**) Second injection, pair-wise comparison of individual data points pre and post injection expressed in log scale, force (in ‘g’ on the left y-axis). (**D**) Individual data points in ratio (in ‘%’), change of pressure pain threshold shown as effect post/pre and bars indicate the median for each group. Effect size is shown as Cohen *d*. NGF, nerve growth factor. CSN: control + saline + NGF; RSS: repeated restraint stress + saline + saline; CNN: control + NGF + NGF; and RSN: repeated restraint stress + saline + NGF. PD: postnatal day. Black circles: control animals, red squares: stressed animals.

In Fig. 3A, a paired plot of the PPT between pre (PD85) and post 1st injection (PD86) is shown for all individual animals. The group that received NGF injection showed a decrease in PPT with medium effect size (CNN: *d* = 0.74) while the groups that received saline showed small or no effects (all *d* < 0.2). In Fig. 3B, the effects of the injections are given as ratios for each group; NGF reduced PPT by about 30%, while the saline effect was about zero.

In Fig. 3C, a paired plot of the PPT between pre (PD90) and post 2nd injection (PD91) is shown for all individual animals. The group that received NGF after adolescent stress showed a decrease in PPT with the largest effect size of all NGF injections (RSN: *d* = 0.87). The group with an NGF injection after handling and saline showed a more pronounced decrease in PPT than expected (CSN: *d* = 0.71), while the group that had received two NGF injections showed only a small effect (CNN: *d* = 0.26), opposite to the expected effect of priming by the 1st NGF injection. In Fig. 3D, the effects of the injections are given as ratios for each group; NGF reduced PPT by about 20–30%, while the saline effect was about zero.

### Paw withdrawal threshold after NGF/saline injections

No effects were observed in the PWT after the injections of NGF/saline (all *d* < 0.2, data not shown). This implies that NGF-induced mechanical hyperalgesia is local to the site of injection in contrast to the widespread stress-induced hyperalgesia.

We also observed no differences in body weight between stressed and control animals during or after the 12 days stress paradigm, nor in adulthood (data not shown). This exhibits that the well-being of the animals was not impaired.

### Microglia contribution to spinal dorsal horn sensitization

To study the contribution of microglia to the sensitization process of the dorsal horn neurons, we performed immunohistochemistry one day after the 2nd injection (PD 91) (see Fig. 1Bb). Results of two-way ANOVAs for the factors state (latent vs. manifest sensitization) and model (adolescent stress vs. two NGF) are summarized in Table 1.

**Table 1:** Comparison of factors state (latent vs. manifest sensitization) and model (adolescent stress vs. two NGF).

Figure panel	Variable	Latent vs. Manifest		Stress vs. NGF		Interaction	
		F_(1,16)_	*p* value	F_(1,16)_	*p***value**	F_**(1,16)**_	*p* value
Figure 4A	Iba-1 intensity	0.207	0.6557	0.756	0.3974	0.467	0.5044
Figure 4B	Number of cells	1.487	0.2404	0.092	0.764	0.189	0.6700
**Morphological states**							
Figure 5A	Surveilling state	1.353	0.2619	4.128	0.0591	9.763	0.0065**
a	superficial dorsal horn	0.639	0.4358	3.740	0.0710	7.330	0.0155*
b	deep dorsal horn	0.486	0.4956	1.187	0.2921	4.560	0.0485*
Figure 5B	Hyper-ramified state	0.001	0.9670	1.644	0.2180	0.042	0.8380
a	superficial dorsal horn	0.207	0.6554	1.862	0.1913	1.246	0.2808
b	deep dorsal horn	0.037	0.8482	1.362	0.2603	0.105	0.7500
Figure 5C	Bushy state	0.132	0.7200	0.047	0.8300	0.064	0.8020
a	superficial dorsal horn	0.284	0.6010	0.688	0.4188	0.003	0.9535
b	deep dorsal horn	0.020	0.8867	0.214	0.6494	0.566	0.4625
Figure 5D	Ameboid state	3.197	0.0920	2.882	0.1080	15.850	0.0010**
a	superficial dorsal horn	3.921	0.0652	1.778	0.2010	16.000	0.0010**
b	deep dorsal horn	0.257	0.6191	3.208	0.0922	6.807	0.0253*
**Morphological state parameters**							
Figure 7A.a	Surveilling: area	0.367	0.5530	0.995	0.3330	9.886	0.0060**
Figure 7A.b	Surveilling: perimeter	1.600	0.2240	3.337	0.0860	5.933	0.0260*
Figure 7A.c	Surveilling: feret’s diameter	1.062	0.3180	0.346	0.5640	7.827	0.0120*
Figure 7A.d	Surveilling: circularity	2.571	0.1280	2.571	0.1280	0.285	0.6000
Figure 7B.a	Hyper-ramified: area	0.103	0.7510	0.096	0.7600	5.114	0.0380*
Figure 7B.b	Hyper-ramified: perimeter	0.024	0.8780	0.236	0.6330	3.557	0.0770
Figure 7B.c	Hyper-ramified: feret’s diameter	0.015	0.9030	1.049	0.3210	7.089	0.0170*
Figure 7B.d	Hyper-ramified: circularity	0.105	0.7490	0.105	0.7490	0.105	0.7490
Figure 7C.a	Bushy: area	2.689	0.1200	5.677	0.0290*	7.411	0.0150*
Figure 7C.b	Bushy: perimeter	7.228	0.0160*	9.266	0.0070**	3.081	0.0980
Figure 7C.c	Bushy: feret’s diameter	7.750	0.0130*	12.900	0.0024**	2.065	0.1700
Figure 7C.d	Bushy: circularity	12.250	0.0030**	6.250	0.0230*	0.250	0.6230
Figure 7D.a	Ameboid: area	1.596	0.2240	0.105	0.7500	2.022	0.1740
Figure 7D.b	Ameboid: perimeter	0.027	0.8700	0.536	0.4740	0.607	0.4470
Figure 7D.c	Ameboid: feret's diameter	0.005	0.9400	0.979	0.3370	0.469	0.5030
Figure 7D.d	Ameboid: circularity	2.462	0.1360	2.462	0.1360	0.005	0.9990

**Figure 4:**
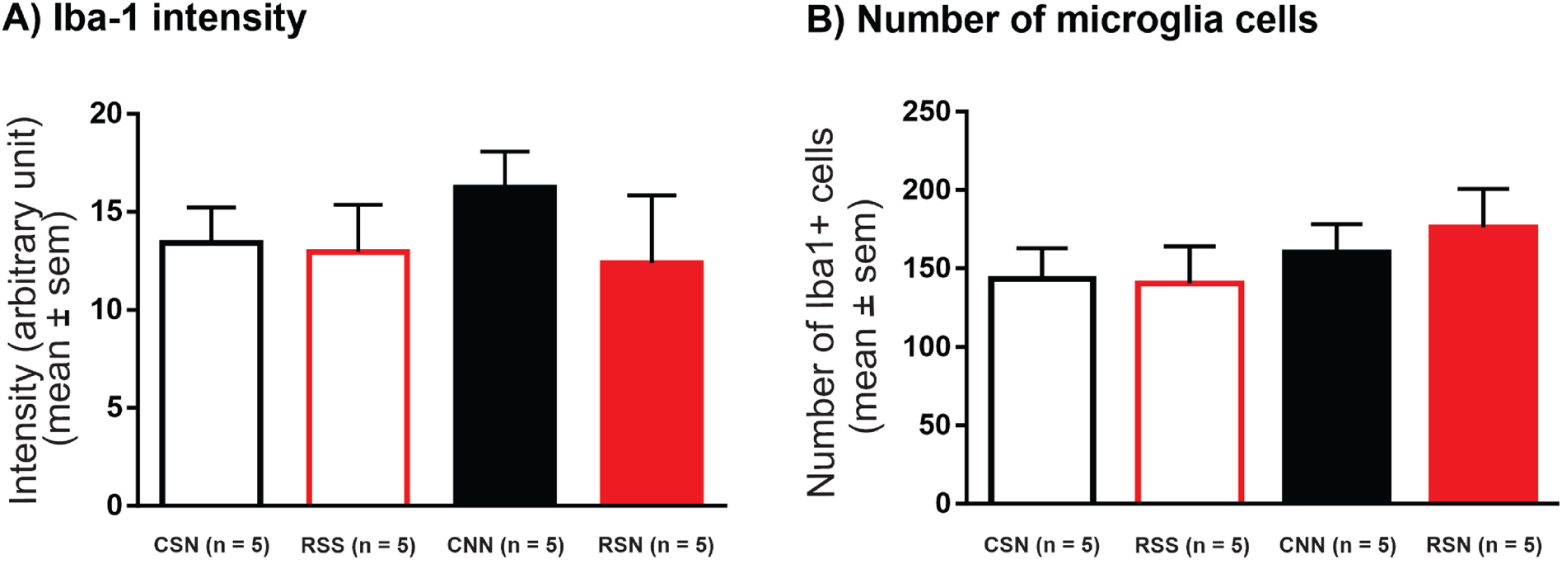
Quantitative analysis of Iba-1 stained microglia cells (ipsilateral side). Repetitive intramuscular NGF injections were associated with a small increase in Iba-1 staining intensity while NGF preceded by adolescent stress was associated with a small increase in number of microglia cells. (**A**) Intensity of Iba-1 stained microglia cells shown in arbitrary unit. Data expressed as mean ± SEM (based on n = 5 animals/group). Intensity and background was measured for each ROI. The background was subtracted and the mean staining intensity was calculated. (**B**) Number of Iba-1 stained microglia cells and data expressed as mean ± SEM. Iba-1 stained microglia cells were counted in each ROI (256 × 256 µm^2^, 20 µm slice thickness, 1.31 nl volume) and the mean was calculated. Iba-1, ionized calcium binding adapter molecule 1; ROI, region of interest; NGF, nerve growth factor. CSN: control + saline + NGF; RSS: repeated restraint stress + saline + saline; CNN: control + NGF + NGF; and RSN: repeated restraint stress + saline + NGF.

**Figure 5:**
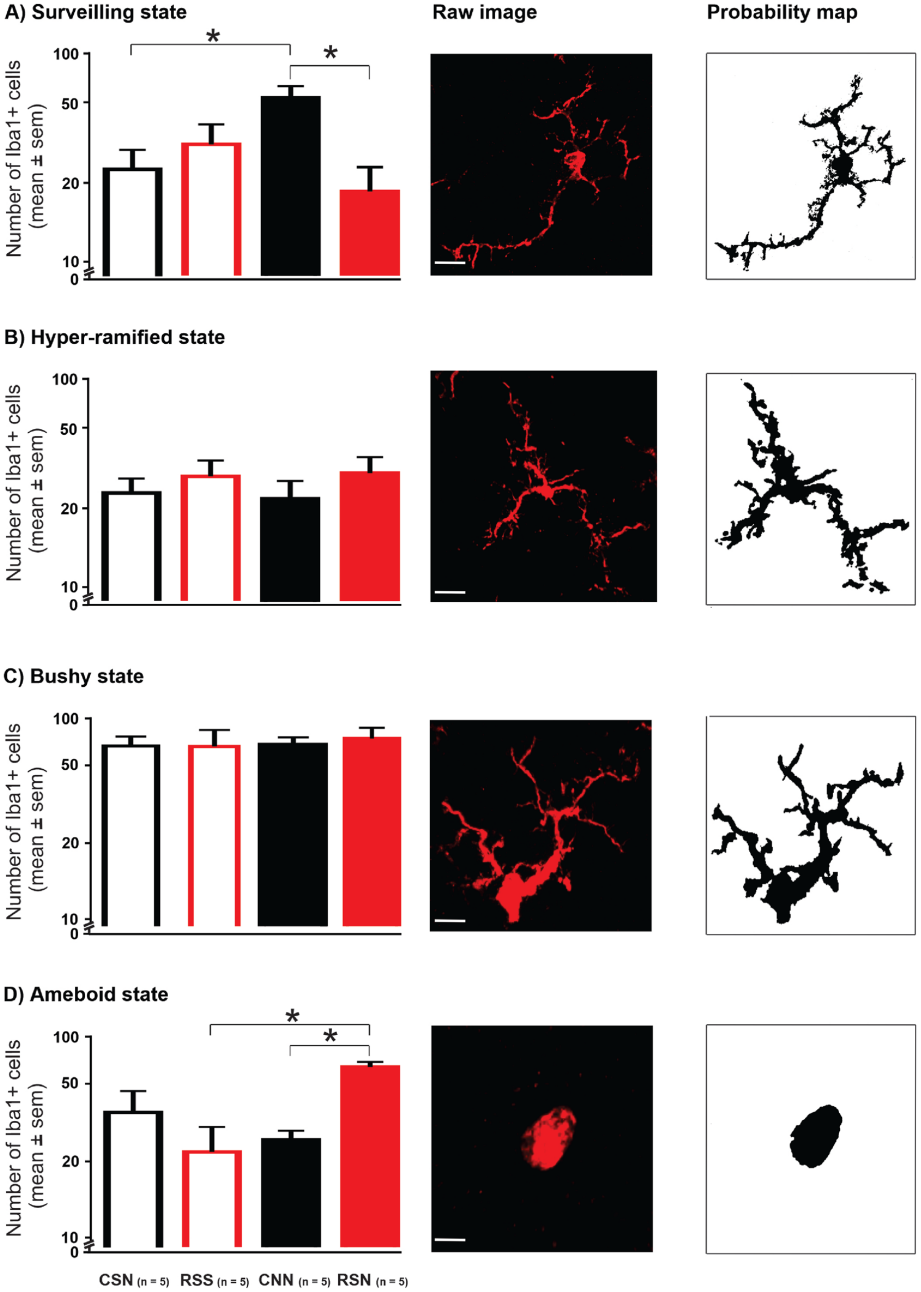
Microglia and its states. Intramuscular NGF injection leads to decreased number of microglia cells in surveilling state and increased number of cells in ameboid state when preceded by repeated restraint stress in early adolescence. Insets show representative raw and probability map images of microglia in all four states (**s**cale bars: 5 µm). (**A**) Number of Iba-1 stained microglia cells in surveilling state. (**B**) Number of Iba-1 stained microglia cells in hyper-ramified state. (**C**) Number of Iba-1 stained microglia cells in bushy state. (**D**) Number of Iba-1 stained microglia cells in ameboid state. Number of microglia cells per state were first averaged across the four ipsilateral ROIs within each animal. Data expressed as mean ± SEM across animals (n = 5 per group); *P* < 0.05: two-way ANOVA followed by Tukey post hoc analysis and significance is indicated by *. Iba-1, ionized calcium binding adapter molecule 1; NGF, nerve growth factor. CSN: control + saline + NGF; RSS: repeated restraint stress + saline + saline; CNN: control + NGF + NGF; and RSN: repeated restraint stress + saline + NGF.

The staining intensity and background was measured for each ROI. The background was subtracted, and the mean staining intensity was calculated (Fig. 4A). The number of cells were counted for each ROI and averaged across the four ipsilateral ROIs within each animal (Fig. 4B), separately per morphological state (Fig. 5A–D). Morphological parameters were obtained for each microglia cell (Fig. 7A–D). The statistics were performed using two-way ANOVA for two main effects and their interaction, significance is indicated by *. ROI, region of interest; Iba-1, ionized calcium binding adapter molecule 1.

### Iba-1 staining intensity and microglia cell numbers

In total, 60 slices of the spinal L2 segment (3 slices/animal; 5 animals/group) were stained for Iba-1, as a marker for microglia. With the small number of animals, we did not find significant main effects nor interactions (Table 1) but small group differences for Iba-1 staining intensity (Fig. 4A) and for number of microglia cells (Fig. 4B). To guide future sample size estimates, we calculated effect sizes for all relevant comparisons (Cohen’s *d*, Table 2). Comparison of groups RSN vs. CSN illustrates potential long-lasting effects of priming by stress; there was no increase in staining but a small increase in number of cells with medium effect size (*d* = 0.7, Table 2B). Comparison of groups CNN vs. CSN illustrates potential long-lasting effects of priming by the first NGF injection; there was an increase with medium effect size for Iba-1 staining (*d* = 0.7) and for number of cells (*d* = 0.7). Comparison of groups RSN vs. RSS illustrates additive effects of the second NGF injection after priming by stress; there was no effect on staining intensity (*d* = 0.0) but an increase with medium effect sizes for number of cells (*d* = 0.6). While this analysis did not reveal major signs of microglia activation, in particular the medium effect sizes are worth further detailed analysis by morphological state of microglia and dorsal horn region.

**Table 2:** Comparison of microglia staining intensity and cell numbers.

Group comparisons	(A) Iba-1 intensity	(B) Number of cells
	Ipsilateral	Ipsilateral
**RSN vs. CSN (stress)**		
*p* Value	*p* = 0.9908	*p* = 0.7075
Cohen *d* (effect size)	0.2	**0.7 ↑**
**CNN vs. CSN (1st injection)**		
*p* Value	*p* = 0.8515	*p* = 0.9438
Cohen *d* (effect size)	**0.7 ↑**	**0.7 ↑**
**RSN vs. RSS (2nd injection)**		
*p* Value	*p* = 0.9984	*p* = 0.6539
Cohen *d* (effect size)	0.0	**0.6 ↑**

The table shows the statistics for (**A**) Iba-1 staining intensity and (**B**) number of microglia cells, on the ipsilateral side of NGF/saline injections. The staining intensity and number of cells were analyzed for each ROI and averaged across the four ipsilateral ROIs within each animal. The statistics were performed using the mean ± SEM (n = 5 animals per group). *P* < 0.05 (two-way ANOVA followed by Tukey post hoc analysis) was considered significant and effect size was calculated using Cohen *d*. The group comparisons shown here are based on two factors: 1) repeated restraint stress (R) and control (C), 2) NGF (N) and saline (S) injections. Iba-1, ionized calcium binding adapter molecule 1; NGF, nerve growth factor. CSN: control + saline + NGF; RSS: repeated restraint stress + saline + saline; CNN: control + NGF + NGF; and RSN: repeated restraint stress + saline + NGF.

### Structural plasticity of Iba-1 stained microglia cells

**Figure 6:**
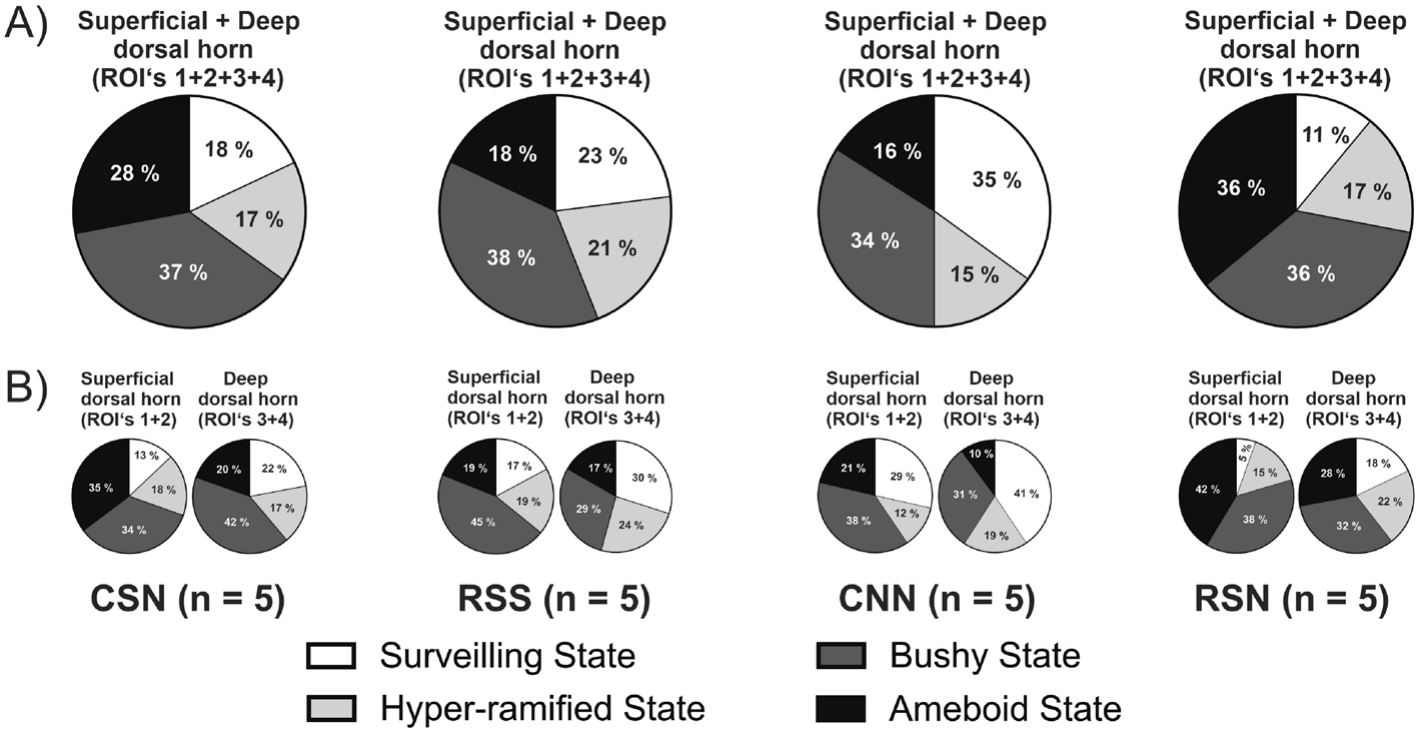
Shifts in proportion of Iba-1 stained microglia cell states. Repeated restraint stress in adolescence and intramuscular NGF in adulthood leads to increased ameboid state microglia cells in the ipsilateral superficial dorsal horn. (**A**) Pie charts illustrate the proportion of Iba-1 stained microglia cells in different states in each treatment group on the ipsilateral (ROI’s 1 + 2 + 3 + 4) dorsal horn. (**B**) Pie charts illustrate the proportion of Iba-1 stained microglia cells in different states in each treatment group on the superficial (ROI’s 1 + 2) and deep (ROI’s 3 + 4) dorsal horn. ROI, region of interest; NGF, nerve growth factor. CSN: control + saline + NGF (721 cells); RSS: repeated restraint stress + saline + saline (707 cells); CNN: control + NGF + NGF (806 cells); and RSN: repeated restraint stress + saline + NGF (886 cells). White: surveilling state; Light grey: hyper-ramified state; Dark grey: bushy state; and Black: ameboid state.

Microglia cells in the ipsilateral dorsal horn were next sorted according to morphological states: surveilling (Fig. 5A), hyper-ramified (Fig. 5B), bushy (Fig. 5C), ameboid (Fig. 5D). Two-way ANOVA revealed significant state by model interactions for surveilling (F_(1,16)_ = 9.763, *P* = 0.0065) and ameboid morphologies (F_(1,16)_ = 15.850, *P* = 0.0010), but not hyper-ramified (F_(1,16)_ = 0.042, *P* = 0.8380) nor bushy morphologies (F_(1,16)_ = 0.064, *P* = 0.8020; Table 1). The ameboid state was most prevalent in group RSN suggesting additive effects of the second NGF injection after priming by adolescent stress; this increase was significant vs. RSS (Fig. 5D; *P* = 0.0044, *d* = 3.7; Table 3A) while there was a large effect size vs. CSN but no significant difference (*P* = 0.1045, *d* = 1.2; Table 3A). The increase in ameboid state was primarily at the expense of the surveilling state (Fig. 5A). These shifts for RSN vs. both RSS and CSN are also illustrated by the pie charts in Fig. 6.

**Table 3:** Comparison of microglia cell numbers in different states.

Group comparisons	A		B			
	Ipsilateral		SDH		DDH	
Number of cells	*p* Value	Cohen *d*	*p* Value	Cohen *d*	*p* Value	Cohen *d*
**RSN vs. CSN (Stress)**						
Surveilling state	*p* = 0.9260	0.4	*p* = 0.8524	**0.6 ↑**	*p* = 0.9923	0.2
Hyper-ramified state	*p* = 0.7863	**0.6 ↑**	*p* = 0.9164	0.4	*p* = 0.7719	**0.6 ↑**
Bushy state	*p* = 0.9757	0.3	*p* = 0.7713	**0.7 ↑**	*p* = 0.9725	0.2
Ameboid state	*p* = 0.1045	**1.2 ↑**	*p* = 0.1295	**1.1 ↑**	*p* = 0.3932	**0.9 ↑**
**CNN vs. CSN (1st injection)**						
Surveilling state	*p* = 0.0359*	**1.8 ↑**	*p* = 0.1017	**1.3 ↑**	*p* = 0.2277	**1.3 ↑**
Hyper-ramified state	*p* = 0.9994	0.1	*p* = 0.6882	**0.6**	*p* = 0.9825	0.2
Bushy state	*p* = 0.9998	0.0	*p* = 0.9865	0.3	*p* = 0.9193	**0.5 ↓**
Ameboid state	*p* = 0.4323	**0.7 ↓**	*p* = 0.5006	**0.8 ↓**	*p* = 0.5253	**1.2 ↓**
**RSN vs. RSS (2nd injection)**						
Surveilling state	*p* = 0.5247	**0.9 ↓**	*p* = 0.5469	**1.0 ↓**	*p* = 0.7421	**0.7 ↓**
Hyper-ramified state	*p* = 0.9980	0.1	*p* = 0.9651	0.4	*p* = 0.9997	0.0
Bushy state	*p* = 0.9711	0.2	*p* = 0.9744	0.2	*p* = 0.9725	0.3
Ameboid state	*p* = 0.0044**	**3.7 ↑**	*p* = 0.0032**	**3.4 ↑**	*p* = 0.1942	**1.0 ↑**

The table shows the statistics for number of microglia cells in different morphological states (**A**) on the ipsilateral side and (**B**) on the superficial and deep dorsal horn on the ipsilateral side. The number of cells were counted for each ROI and averaged across ROIs within each animal. The statistics were performed using the mean ± SEM. *P* < 0.05 (two-way ANOVA followed by Tukey post hoc analysis) was considered significant indicated by * and effect size was calculated using Cohen *d*. The group comparisons shown here are based on the factors: 1) repeated restraint stress (R) and control (C), 2) NGF (N) and saline (S) injections. ROI, region of interest; SDH, superficial dorsal horn; DDH, deep dorsal horn; Iba-1, ionized calcium binding adapter molecule 1; NGF, nerve growth factor. CSN: control + saline + NGF; RSS: repeated restraint stress + saline + saline; CNN: control + NGF + NGF; and RSN: repeated restraint stress + saline + NGF.

Unexpectedly, significant differences were found between the two groups predicted to be both in a state of manifest sensitization: CNN and RSN. The ameboid state was more frequent in RSN than CNN (Fig. 5D; *P* = 0.0050), accompanied by a decrease in surveilling state (Fig. 5A; *P* = 0.0105) indicating larger effects when NGF given as the second injection was primed by adolescent stress rather than by a first NGF injection in adulthood. This unexpected lack of priming effect by a first NGF injection was also illustrated by higher rather than lower number of cells in the surveilling state in CNN than CSN (Fig. 5A; *P* = 0.0359; Table 3A).

To guide future sample size estimates, we again calculated effect sizes for the relevant comparisons (Cohen’s *d*, Table 3). Group RSN exhibited a medium increase in hyper-ramified state (*d* = 0.6) and a large increase in ameboid state vs. CSN (*d* = 1.2), illustrating mild long-lasting effects of priming by stress that had also led to a small increase in number of cells with medium effect size (*d* = 0.7, Table 2B). Group CNN exhibited a medium decrease in ameboid state (*d* = 0.7) and a large and significant increase in surveilling state vs. CSN (*d* = 1.8), illustrating the opposite effect of what had been expected from priming by the first NGF injection; the increases in Iba-1 staining and number of cells (both *d* = 0.7, Table 2) were hence due to increases in surveilling microglia. Group RSN exhibited a very large and significant increase in ameboid state (*d* = 3.7) and a large decrease in surveilling state vs. RSS (*d* = 0.9), underlining that the additive effect of the second NGF injection after priming by stress was accompanied by microglia activation that explains the medium increase in number of cells (*d* = 0.6, Table 2B).

Table 1 also shows that the changes in numbers of microglia per morphological state were more pronounced in superficial (surveilling: F_(1,16)_ = 7.330, *P* = 0.0155; ameboid: F_(1,16)_ = 16.0, *P* = 0.0010) than deep dorsal horn (surveilling: F_(1,16)_ = 4.560*, P* = 0.0485; ameboid: F_(1,16)_ = 6.807*, P* = 0.0253). The pie charts in Fig. 6B, plotted based on the relative numbers further illustrate that shifts from surveilling towards ameboid state for RSN vs. both RSS and CSN occurred in a more pronounced fashion in the superficial than deep dorsal horn (see Table 3B for statistics and effect sizes based on absolute microglia cell numbers). The relevance of ameboid state in priming by adolescent stress is supported by a correlation analysis of behavior vs. microglia state across animals per experimental group: relative drop in PPT correlated with number of cells in ameboid state only in RSN (r = 0.701, *p* < 0.001) and not in the other three groups.

These findings, especially the ameboid state microglia, suggest that repeated restraint stress in early adolescence primes the spinal DHNs for a longer period so they are easily susceptible to manifest sensitization when presented with a second hit (NGF) in the adulthood phase.

### Morphological evaluation of Iba-1 stained microglia cells

**Figure 7:**
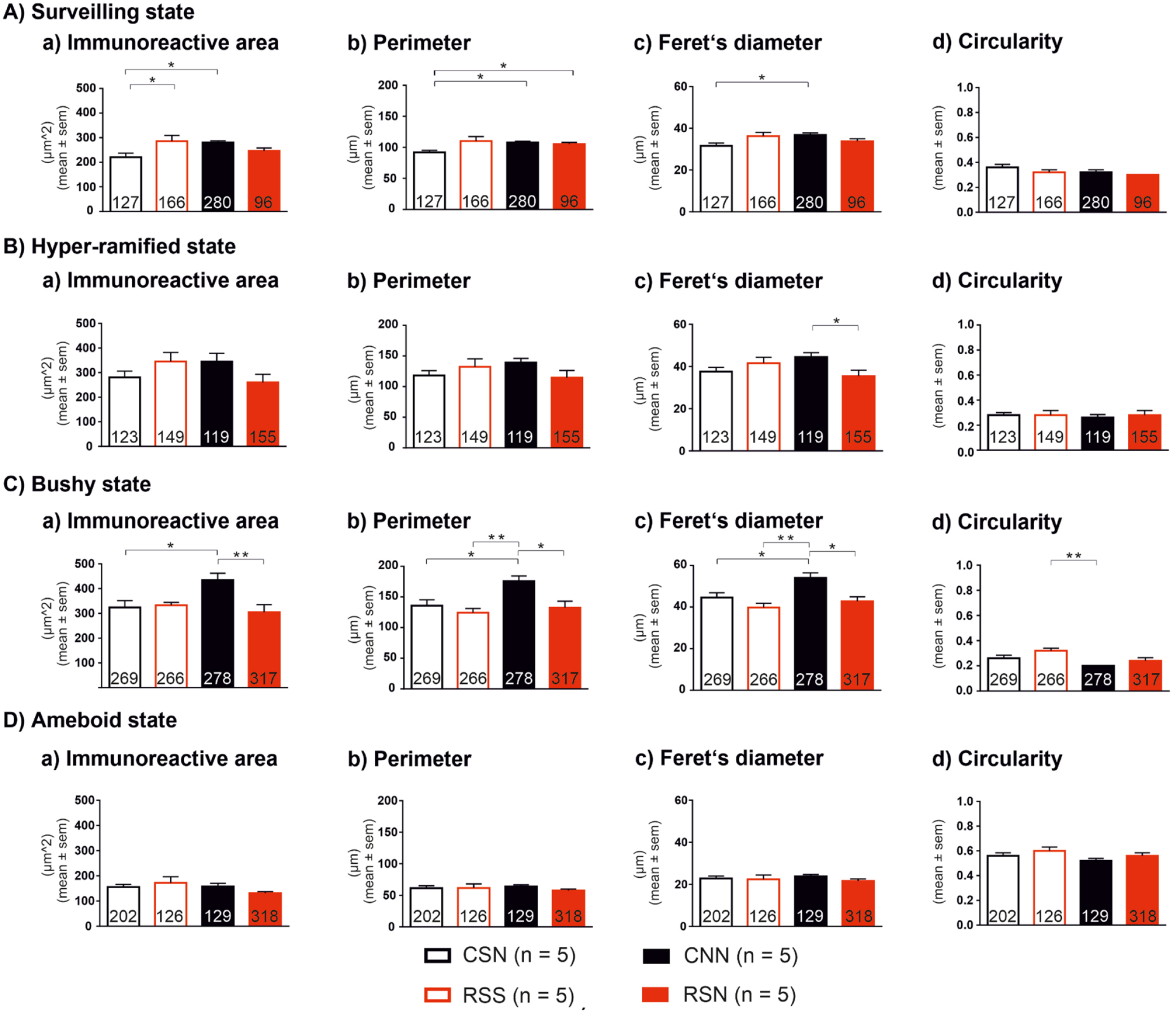
Morphological evaluation of Iba-1 stained microglial cells. For each of the four morphological states (**A**–**D**), four quantitative measures (**a**–**d**) were evaluated for each of the four experimental groups (CSN, RSS, CNN, RSN). Total numbers of microglia cells evaluated are given inside respective bars; the ranges of numbers of cells per animal are: (**A**) Surveilling state: CSN: 16–51, RSS: 13–53, CNN: 34–84, RSN: 1–42; (**B**) Hyper-ramified state: CSN: 17–39, RSS: 15–45, CNN: 13–42, RSN: 17–47; (**C**) Bushy state: CSN: 31–98, RSS: 14–144, CNN: 28–87, RSN: 34–128; (**D**) Ameboid state: CSN: 18–80, RSS: 8–41, CNN: 20–35, RSN: 49–72. Morphological parameters per microglia state were first averaged across all cells within each animal. Data expressed as mean ± SEM across animals (n = 5 per group). *P* < 0.05: two-way ANOVA followed by Tukey post hoc analysis or *U*-test of Mann and Whitney and significance is indicated by *. NGF, nerve growth factor. CSN: control + saline + NGF; RSS: repeated restraint stress + saline + saline; CNN: control + NGF + NGF; and RSN: repeated restraint stress + saline + NGF.

A detailed analysis on the morphology of individual microglia cells was performed to assess potential additional differences between the four groups of animals beyond the numbers of these cells in the four morphological states (Fig. 7). We found significant state by model interactions for surveilling and less so for hyper-ramified states (Table 1). Both main effects were significant for bushy state, but post-hoc Tukey tests did not support this and argue more for an interaction as well. There were no main effects nor interactions for ameboid state, indicating that microglia in this state had uniform shapes and differ only in their numbers. We summarize these findings again according to the planned group comparisons.

Group RSN exhibited similar immunoreactive area, perimeter, Feret’s diameter and circularity as group CSN across all four morphological microglia states except for the perimeter in surveilling state (Fig. 7Ab; *P* = 0.0439). This illustrates that major morphological alterations did not accompany the observed mild long-lasting effects of priming by stress, which were: increase in total number of cells with medium effect size (*d* = 0.7, Table 2B) especially in hyper-ramified state (*d* = 0.6, Table 3A) and a large and significant increase in cells in ameboid state (*d* = 1.2, Table 3A).

Group CNN exhibited larger immunoreactive area (Fig. 7Aa; *P* = 0.0442), perimeter (Fig. 7Ab; *P* = 0.0492) and Feret’s diameter (Fig. 7Ac; *P* = 0.0370) than group CSN for surveilling state. In bushy state, CNN exhibited larger immunoreactive area (Fig. 7Ca; *P* = 0.0324), perimeter (Fig. 7Cb; *P* = 0.0289) and Feret’s diameter (Fig. 7.C.c; *P* = 0.0394) than group CSN. This illustrates that priming by the first NGF injection had enlarged the microglia which explains the increases in Iba-1 staining (*d* = 0.7, Table 2) although the increase in number of cells had been due to increases in cells in surveilling state only.

We also observed unexpected significantly increased immunoreactive area (Fig. 7Ca; *P* = 0.0113), perimeter (Fig. 7Cb; *P* = 0.0175) and Feret’s diameter (Fig. 7Cc; *P* = 0.0126) in CNN vs. RSN, indicating larger priming effects on the morphological parameters of the bushy state microglia by the first NGF than by adolescent stress.

Group RSN exhibited similar immunoreactive area, perimeter, Feret’s diameter and circularity as group RSS across all four morphological microglia states. This lack of morphological alterations explains why the additive effect of the second NGF injection after priming by stress had led to a very large increase in ameboid state (*d* = 3.7, Table 3A) and a large decrease in surveilling state vs. RSS (*d* = 0.9, Table 3A), despite only a medium increase in number of cells (*d* = 0.6, Table 2B).

## Discussion

The present study shows that repeated restraint stress across adolescence induces long-lasting muscle (PPT) and short lasting cutaneous (PWT) hyperalgesia. In contrast, i.m. NGF injection in adulthood led to lowered PPT (local) but not PWT (remote). These findings suggest that NGF injection is a model of local back pain, whereas adolescent stress induced signs of widespread hypersensitivity as in fibromyalgia. The repeated NGF without preceding stress in Wistar rats did not show the local nociceptive priming effects known from Sprague–Dawley rats, but there was a slight priming effect of adolescent restraint stress on sensitization by subsequent i.m. NGF injection in adulthood.

Analysis of the immunohistochemistry data addressed three predictions: 1. potential long-lasting effects of priming by stress (RSN vs. CSN); we observed only minor differences two months after stress, which consisted of an increase in number of microglia cells in particular in ameboid state without major morphological changes within microglia state. 2. potential effects of priming by the first NGF injection (CNN vs. CSN); we observed a moderate increase in Iba-1 staining intensity accompanied by an increase in microglia in surveilling state, which may indicate a residue of proliferation induced by the first NGF injection, and larger individual cells in both surveillant and bushy states, which may be the response to NGF given as the second injection five days later. 3. additive effects of the second NGF injection after priming by stress (RSN vs. RSS); we observed an increase in number of microglia cells in particular in ameboid state without major morphological changes.

Thus, nociceptive priming by preceding NGF that was characterized by increase in microglia in surveilling state with larger cell sizes was different from priming by preceding stress that was characterized by a shift towards the ameboid state that correlated with behavioral sensitization but with unchanged morphology of individual microglia cells.

### Repeated restraint stress in adolescence alters pain sensitivity into adulthood

We observed decreased thresholds to pressure pain (PPT) in the lower back and for paw withdrawal (PWT) of stressed animals shortly after the cessation of the stress paradigm (PD34). These data indicate that adolescent stress induces acute sensitization to nociceptive inputs from two locations (low back, paw) and two tissues (muscle, skin), i.e., signs of widespread hyperalgesia as known in humans suffering from fibromyalgia^35^. Previous studies from our group on effects of stress in adult rats had found smaller behavioral effects^20^, although electrophysiological signs of primed spinal neurons were observed (increased resting activity)^19^. These findings suggest that stress in adolescence induces manifest widespread hypersensitivity, while stress in adulthood only induces latent sensitization. This underlines the importance of timing of the stressor^36,37^ in critical windows of enhanced neuroplasticity during which adverse events have long-term effects on pain sensitivity, physical and mental well-being^38^.

The difference in axial muscle PPT between stressed and non-stressed groups remained over time, while the difference for distal cutaneouos PWT declined. In another study on early-life stress (neonatal limited bedding), the rats in adulthood also developed mild muscle hyperalgesia^39^. These findings suggest that low-back muscles may be particularly sensitive to widespread pain sensitization by adolescent stress, which is consistent with reports that adverse childhood experiences in humans predispose to the development of chronic widespread myofascial pain including the lower back^7^.

### The roles of microglia in central sensitization of the nociceptive system

Activated microglia play a key role in inflammatory CNS reactions and in neural plasticity^40^. Microglia can be activated by a feedback loop through the release of fractalkine (CX3CL1) from primary afferent terminals and DHNs, binding to CX3CR1 receptors on microglial cells^41,42^. We had found in our NGF-induced myofascial low-back pain model that blocking microglial activation prevented spinal latent sensitization while blocking astrocyte activation reversed it^26^. Blocking fractalkine signaling by neutralizing antibodies also prevented NGF-induced sensitization supporting the critical role of the CX3CL1-CX3CR1 pathway^27^. Thus, neuroinflammatory microglia-neuron interactions are required to sensitize dorsal horn neurons in our NGF-induced myofascial low-back pain model^26,27^. As a sign of spinal sensitization, PPT sensitivity increased and DHN acquired new receptive fields in deep tissues of the low back and lower limb^11^. Other studies have shown that heterosynaptic long-term sensitization of adjacent unstimulated neurons in the spinal cord is due to gliogenic spread^28^.

The activation of microglia is highly complex, heterogeneous and stimulus dependent. Upon insult, microglia change their morphology and function^43,44^ and once activated may eliminate toxic synaptic elements^45,46^. Since our behavioral findings show increased sensitivity to PPT of the low back muscles, we think the ameboid shaped microglia in our results are in an activated, pro-inflammatory state. Our finding that the proportion of these activated microglial cells was higher in the superficial dorsal horn compared to the deep dorsal horn (Fig. 6B) shows that adversity in adolescence plays an important role in long-term priming processes in the superficial dorsal horn, which is the first synaptic relay in nociceptive pathways^47^.

### Manifest vs. latent sensitization induced by adolescent stress and NGF

Priming by stress during adolescence by itself was strong enough to induce long-lasting sensitization to nociceptive input from the lower back in adulthood (manifest sensitization). A second hit by mild nociceptive input induced by NGF in adulthood further exacerbated pain sensitization, suggesting a small additive effect of nociceptive input on an additional state of stress-induced manifest sensitization.

In contrast, we did not find long lasting nociceptive priming by the first NGF injection in rats that had not been stressed. Although the initial NGF injection increased sensitivity to pressure pain, this hypersensitivity returned to baseline on day two (data not shown), and the second NGF injection did not lead to further increases in sensitivity. This finding in Wistar rats is different from our previous studies in Sprague–Dawley (SD) rats^11,26^, suggesting strain differences in pain sensitization. Such strain differences have been investigated extensively across inbred mouse lines^48^ but much less for outbred rat strains. Wistar rats develop about 50% less mechanical hyperalgesia in an inflammatory pain model (CFA) than Sprague–Dawley rats^49^. One possible explanation is a stronger habituation to the test–retest setting by Wistar rats, i.e., the sensitization-habituation balance may be shifted towards habituation. Such a shift is known for acoustic startle responses^50^. In turn, Wistar rats are more vulnerable to the effects of stressors^51^. This could explain why Wistar rats showed significant drop in PPT after stress but only medium effect sizes after NGF administration. While Wistar rats may be a good model for humans with adverse childhood experiences due to their pronounced stress sensitivity^52^, Sprague–Dawley rats may better reflect nociceptive priming in non-stressed humans.

In immunohistochemistry we had predicted similar effects of nociceptive priming by stress (RSN vs. CSN) and by mild nociceptive inputs (CNN vs. CSN), i.e., commonalities between latent sensitization on one hand (CSN and RSS) and manifest sensitization on the other hand (RSN and CNN). Instead, we found differential effects on microglia. Adolescent stress increased the number of microglia in the ameboid state (proliferation accompanied by activation, moderate effect sizes may be explained by the 60 days delay between stress and tissue collection). Repeated NGF injections without adolescent stress led to an increase in number of microglia in surveilling state and significantly increased cell sizes, possibly indicating that proliferation may have been a residue of the first NGF injection and that there was habituation rather than sensitization of activation by NGF, consistent with the behavioral data.

### Potential mechanisms for long-term sensitization by adolescent stress

Adolescent stress by itself immediately induced a pronounced and widespread hypersensitivity to painful stimuli. One possible mechanism could be activation of an excitatory pathway from the medial hypothalamus to rostral ventral medulla oblongata^53,54^ and onward to the DH in a phenomenon called ‘stress-induced hyperalgesia’; increased descending facilitation or decreased descending inhibition could as well enhance the excitability of DHNs^53^. Crosstalk between microglia and neurons in the spinal cord contributes to stress-induced hyperalgesia^55^. Thus, microglia are likely involved in short term sensitization on PD 34. Mechanisms of long-term sensitization up to PD90 are more elusive, but they are required to explain the clinical phenomenon that childhood adversities prime for myofascial pain. Memory processes are known for the nervous system and the immune system. Early-life events modulate normal learning-dependent cytokine activity within the hippocampus via a specific and enduring impact on brain microglial function^56^. Moreover, plasticity of the immune response of neonate microglia compared with microglia derived from mature and aged mouse brain is more pronounced^57^. We thus propose that long-term priming by adolescent stress critically involves long-term memory in spinal microglia. Given the long delay after the actual stress (60 days) we consider the increase in number of cells (moderate effect size) to be a subtle sign of long-lasting priming of microglia by adolescent stress that then more readily shifted to ameboid state after NGF challenge.

### Limitations

First, Wistar rats were chosen because of their known sensitivity to stress^51,52^ and the observed behavioral effects of stress included manifest sensitization. Future studies could use milder adolescent stressors such as social isolation that only induce latent sensitization, to be more sensitive to additive effects of subsequent mild nociceptive input by NGF injection^20^.

Second, we lack a pure negative control (control + saline + saline). Considering the 3R principle, we did not repeat this control experiment, because former electrophysiological studies had found no differences between two saline injections and latent sensitization by one NGF^11,26^. Absence of this treatment group limits the interpretation of some unexpected findings in our current study.

Third, due to the necessity for habituation to its setup, we were unable to obtain a baseline measurement for the PWT. This limits our conclusions on maturational changes with age but not the effects of adolescent stress.

Finally, we assessed only male rats in this study for comparison with previous work in this model^11,58,59^. One laboratory has already demonstrated that NGF-induced myofascial pain and hypersensitivity occur in female rats^60,61^, but the mechanisms of hypersensitivity in neuropathic pain are known to differ between females and males^62^. Therefore, sex differences in mechanisms of pain hypersensitivity induced by adolescent stress followed by NGF injection in adulthood are an important future research topic.

## Conclusions

The observation that repeated restraint in adolescence induced long lasting muscle and short-lasting cutaneous hyperalgesia suggests that adolescent stress may predispose for widespread pain sensitization and that low-back muscles may be particularly sensitive to this effect. This is reminiscent of known effects of stress due to adverse childhood experiences in humans. In addition, stressed rats were primed for enhanced manifest sensitization by a subsequent NGF, local to the site of injection. This is indicated by a drop in PPT of the low back muscles, decreased number of spinal microglia in surveilling state, and increased number of cells in the ameboid state. In contrast, repeated NGF injections without stress only increased cell size in surveilling and bushy microglia states without behavioral sensitization. This novel rodent model shows that adolescent stress is a risk factor in the development of chronic LBP in adulthood and that morphological changes in microglia are signs of spinal mechanisms involved.

## Materials and methods

### Experimental animals and housing conditions

Twenty-four Wistar HAN rats were used in the study. The study was done in male animals only, in order to be able to compare our findings with previous studies on NGF-induced myofascial pain in rats and mice that were also limited to males^11,20,26,27,58,59^. Animals arrived from ENVIGO (Netherlands) on postnatal day (PD) 21 and were housed in groups of four in standard macrolon cages (length, width, height: 55 × 35 × 20cm). Animals had free access to food and water *ad* libitum and were kept on a normal 12 h light/dark cycle. Animals were briefly (2 h) acclimatized to their new home cage in the housing room and 1 h to the experimental procedure room on arrival. All experiments were conducted in the inactive phase of the animals. All experimental procedures were approved by the Committee on Animal Care and Use (Regierungspräsidium Karlsruhe, Germany; permission number: AZ 35-9185.81/G-7/19) and were carried out in accordance with German Law on the protection of animals and ethical proposals of the International Association for the Study of Pain. The study is reported in accordance with ARRIVE guidelines. One animal died (day three of stress paradigm, PD 24) and all data from this animal were excluded from further analyses. This work is preregistered online in Open Science Framework (OSF|Impact of ESA on latent and manifest sensitization of dorsal horn neurons to myofascial input from the low back in rats).

### Repeated restraint stress

The stress paradigm was induced during the early adolescent phase. Restraint stress was induced as previously described^19,20^ by placing animals in a narrow restrainer (inner length 15 cm; inner height 4 cm) for 1 h daily on 12 consecutive days (Fig. 1A). The control animals were handled on 12 consecutive days (transported to the laboratory and manipulated by hand) by the same experimenter (ADG). Body weight was measured every day immediately before the start of the stress paradigm or handling. Since our first study with restraint stress^19^ had verified elevated fecal levels of corticosterone metabolites, we did not monitor endocrinological signs of stress here, but animals were monitored for signs of distress such as vocalization, struggling during restraint (escape movements), urination, and/or defecation during the stress paradigm^20^.

### Injection of nerve growth factor or saline

As second and third interventions in adulthood, animals received intramuscular injections of saline or NGF at two different time points with an interval of 5 days (see Fig. 1A). Nerve Growth Factor (0.8 µM; NGF, human recombinant, Calbiochem, MERCK, Germany) was dissolved in phosphate buffer saline (PBS: pH of the NGF solution 7.2–7.3) and 50 µl injections were made into the left multifidus muscle 3 mm lateral to the spinous process at vertebral level L5^11,26^. The NGF concentration used is not painful but induces hyperalgesia when intramuscularly injected in animals or humans^11,16,63,64^. Injections of isotonic saline (50 µl, 0.9%) served as control. No visible signs of muscle inflammation were observed after the injections of neither NGF nor saline^11^.

### Treatment groups and planned comparisons

We aimed to compare latent vs. manifest sensitization induced by injections of NGF (as a model of mild nociceptive input) or adolescent restraint (as a model of childhood adversities) or both. We applied combinations of three interventions: restraint stress or control handling (R or C) followed by two injections of NGF or saline (N or S) or both. We assessed four groups (out of eight possible combinations): RSS and CSN were predicted to induce latent sensitization (microglia activation but no behavioral signs), while RSN and CNN were predicted to induce manifest sensitization (microglia activation plus signs of hyperalgesia). Animals were randomly assigned to the four treatment groups and each cage had two control and two stress rats.

**RSS: Repeated restraint stress + Saline + Saline**. Repeated restraint stress (n = 6) was induced on 12 consecutive days for 1 h every day in a narrow plastic tube. These animals received 2 saline injections 5 days apart.

**CSN: Control + Saline + Nerve growth factor.** The control animals (n = 6) were handled on 12 consecutive days without repeated restraint stress. These animals received saline (vehicle) as their 1st injection followed by NGF at an interval of 5 days.

**RSN: Repeated restraint stress + Saline + Nerve growth factor.** Repeated restraint stress (n = 5) was induced on 12 consecutive days for 1 h every day in a narrow plastic tube. These animals received saline as their 1st injection followed by NGF at an interval of 5 days.

**CNN: Control + Nerve growth factor + Nerve growth factor.** The positive control animals (n = 6) were handled on 12 consecutive days without repeated restraint stress. These animals received 2 NGF injections at a 5-day interval in adulthood.

There were three comparisons of interest: RSN vs. CSN to illustrate potential long-lasting effects of priming by stress; CNN vs. CSN to illustrate potential long-lasting effects of priming by the first NGF injection, RSN vs. RSS to illustrate additive effects of the second NGF injection after priming by stress.

### Behavioral tests

Behavioral tests were performed to assess for mechanical hyperalgesia of the low back muscles (Pressure pain threshold) local to the site of NGF/saline injections and remote hind paw skin (Paw withdrawal threshold). All behavioral measurements were done by the same blinded experimenter (SKS) and the experimental setups were cleaned with 70% ethanol between animals. Our major focus in this study is on the side ipsilateral to the side of the NGF/saline injections and for this reason, only the data of the left lower back and left hind paw are presented and discussed in this article.

### Pressure pain threshold

To test for local mechanical hyperalgesia the pressure pain threshold (PPT) of the low back was determined with an electronic Von Frey anaesthesiometer (LIFE SCIENCE INSTRUMENTS, Woodland Hills, CA, USA). A blunt tip with an area of 3.46 mm^2^ was pressed with increasing intensity to the MF muscle through the intact skin at the vertebral level L5. With the blunt tip, mainly nociceptors in deep tissues but not in the skin were excited^65^. The PPT was defined as the minimum pressure intensity that is required to elicit a pain-related reaction (vocalization, withdrawal, and escape movements). PPT was tested before and after stress, and in conjunction with intramuscular injections (red line in Fig. 1A).

### Paw withdrawal threshold

To test for remote mechanical hyperalgesia, the paw withdrawal threshold to punctate mechanical stimuli was performed in both distal hind limbs, after completing PPT measurements on the low back. For this purpose, the electronic von Frey esthesiometer was equipped with a rigid cylindrical tip (diameter 0.8mm^2^). The animals were placed into a Plexiglas box (length, width, height: 20 × 10 × 14 cm) with a metal grid as a base for a further 30 min of habituation. Increasing punctate pressure was applied to the plantar region of both hind paws (ipsilateral to the injection site followed by contralateral) until the rat withdrew it. The paw withdrawal threshold (PWT) is calculated as the mean of five independent measurements. Before the first measurement on PD 36, the animals had to be habituated to the metal grid table on two consecutive days for 45 min each. Therefore, PWT was first tested on PD36 after habituation on PD34 and PD35 (red line in Fig. 1A).

### Perfusion and tissue processing

One day after the 2nd injection of NGF or saline (see Fig. 1A), animals were euthanized with an overdose of thiopental sodium i.p. (Trapanal, INRESA GmbH, Germany) and transcardially perfused with 4% paraformaldehyde (PFA) in 0.1 M PBS. After the perfusion, a laminectomy was performed and the spinal segment L2 was removed and stored in 10% sucrose solution in 0.1 M PBS at 4 °C for 1 day. One day before freezing, spinal segments were transferred to a 30% sucrose solution in 0.1 M PBS at 4 °C. The spinal segment L2 (Fig. 1Ba) was chosen since previous experiments had found that segment L2 receives most of the input from low back muscles at spinal level L5^66,67^. Neuroanatomical studies confirmed that afferents from low back structures project to dorsal root ganglia located several segments cranially relative to the segmental location of their receptive fields^68^. Twenty µm thick cross-sections of the spinal segment were made on a cryostat (Cryostat NX70, THERMO FISCHER SCINETIFIC INC., USA) and mounted on glass slides. For immunohistochemistry in each treatment group, tissues from five animals were randomly chosen, yielding 60 sections for analysis (4 groups × 5 animals × 3 sections within L2).

### Immunofluorescence labeling, image acquisition and image processing

#### Immunofluorescence labeling

Structural changes of microglial cells were visualized by ionized calcium-binding adapter molecule 1 (Iba-1) immunohistochemistry. Iba-1 is a protein that is expressed in microglia and is upregulated under painful conditions^30^. The sections were first incubated in 10% Roti-block (CARL ROTH, Germany) at room temperature for 1 h followed by rabbit anti-Iba-1 polyclonal antibody (1:1000; ABCAM, United Kingdom) at room temperature for 16 h. Later, sections were incubated in secondary antibodies, Cy3—conjugated goat-anti-rabbit IgG antibody (1:500; JACKSON IMMUNORESEARCH, USA) at room temperature in darkness for 4 h. Tissues were washed 3 times in PBS for 5 min each and mounted with Roti mounting medium (CARL ROTH, Germany).

#### Image acquisition

Digitized images were obtained using a confocal laser-scanning microscope (LEICA TCS SP8 AOBS, Wetzlar, Germany). Immunofluorescence of the Cy3—conjugated secondary antibody was detected by a DPSS—laser (561 nm) (LEICA MICROSYSTEMS, Germany). First, an image using 10 × objective lens was captured to get an overview of the entire L2 section (Fig. 1Bb left) with a computer-based imaging software LAS X Navigator (LEICA, Germany). For analysis of single cell morphology including all processes, three-dimensional images of the dorsal horn over 20-μm z-axis with a step size of 1.0 μm were acquired using a 40 × oil immersion objective lens (Fig. 1Bb middle, Raw image).

### Image processing

For quantitative measurements, 8 regions of interest (ROIs) were selected in each of the 60 sections. They were located in the dorsal horn ipsilateral (Ip.) and contralateral (Con.) to the injection site (Fig. 1Ba). At each side, 4 ROIs of 256 μm × 256 μm were defined with the help of IMAGEJ software (ImageJ bundled with Java 1.8.0_172; NIH, USA) and each ROI was then saved as an individual .TIF file (Fig. 1Bb middle, Raw image). Two were located medial and lateral in the superficial dorsal horn (mainly laminae I and II); and two in the deep dorsal horn one more dorsally (laminae III–IV) and a second ventrally (laminae IV–V; see Fig. 1Ba). For Iba-1 staining intensity, each ROI was measured, and the background was subtracted.

In some microglia cells the processes that belonged to the somata were not connected and some processes did not show any neighboring soma. We used the “ILASTIK” software (version 1.3.3post3 (2020-05-03)) and its pixel classification workflow^69^ to identify microglia somata and their associated processes. The pixel classification assigns labels to pixels based on pixel features and user annotations. Ten random images were selected, and two semantic classes were defined as ‘connect processes’ and ‘remove processes’. For each class, examples were provided by the blinded experimenter. For each pixel of the image, “ILASTIK” then estimates the probability that the pixel belongs to either of the above-mentioned semantic classes. Once the training was sufficient to distinguish connect and remove processes, all 480 images were loaded for pixel classification. The resulting probability maps were exported as. TIF flies and used for further analyses using IMAGEJ (Fig. 1Bb right, Probability map). This way each z-stack 3-D image was condensed into a 2-D image.

### Microglia states classification and morphological evaluation

The different morphological states of microglia were classified as defined in^31^. In each ROI, the Iba-1 positive cells were manually counted by the blinded experimenter (SKS), and the morphology was differentiated into:

1. *Surveilling state:* characterized by small cell bodies, long, thin and highly motile processes^70^.
2. *Hyper-ramified state (intermediate):* characterized by long and thick processes^31^.
3. *Bushy (activated):* characterized by enlarged and darkened soma, thick and less ramified processes or rod shaped with long and polarized processes^71,72^.
4. *Ameboid state:* microglia show an amoeboid shape with enlarged and densely stained soma with few or no processes^73^.

### Morphological evaluation of microglia

For each animal and each of the four microglia states, the following parameters were averaged across all Iba-1 stained microglia cells (IMAGEJ software):

Immunoreactive area of a single cell: is the number of pixels selected by ‘ILASTIK’ (calibrated to µm^2^). The immunoreactive area is expected to increase with the hypertrophism of cells due to activation, soma enlargement, and sprouting of new ramifications^74^.

Perimeter of a single cell: was calculated based on the outline length (boundary length) of the immunoreactive area and is expressed in µm. The perimeter is expected to be higher in ramified cells^74^.

Feret’s diameter of a single cell: is the longest distance between two parallel lines perpendicular to that distance (the longest distance) and drawn at boundary of the immunoreactive area. Feret’s diameter is expected to increase with cell hypertrophism and new ramification elongation^74^.

Circularity of a single cell: is calculated using the formula 4π x (area/perimeter^2^) and varies from ‘0’ (linear polygon) to 1 (perfect circular object). The cells become more circular in activated and ameboid state because of fewer or no ramification^74^.

## Data analysis

The experimenter doing the behavioral experiments and immunohistochemical microglia analysis (SKS) was blinded to the treatments the animals had received by another experimenter (ADG).

Before calculations, the data of PPT and PWT were transformed into decadic logarithms to achieve secondary normal distribution^75^, since previous data obtained in larger cohorts provided solid evidence for the log-normal distribution of PPT and other psychophysical data^76^. For data presentation in figures, PPT and PWT values were normalized to the control group as ratios (mean R.stress/mean Control) and presented as percentage changes. Data are presented as mean and SEM (based on number of animals per group n = 5 or 6).

Statistical analyses were performed with GRAPHPAD PRISM (version 6) by using analysis of variance (ANOVA) followed by Tukey post hoc analysis, as indicated in figure legends. A normal distribution of the data was tested with the Kolmogorov–Smirnov test and, when data did not fit the rules of parametric analysis, comparisons were performed with the Mann–Whitney *U* test. Statistical significance was considered with an alpha level of 0.05 or lower (*P* < 0.05, two-tailed). The PPT and PWT data after NGF/saline injections are shown as individual paired values (Fig. 3A and C) and effect sizes were determined using Cohen *d* (difference in means divided by pooled SD). An effect size > 0.2 was considered as ‘small’, > 0.5 as ‘medium’, and > 0.8 as ‘large’^77^.

## Data Availability

The data sets generated and analyzed during the current study are available from the corresponding author on reasonable request.
